# ﻿Taxonomic study of the genus *Kuvera* Distant, 1906 (Hemiptera, Fulgoromorpha, Cixiidae) with descriptions of two new species from China

**DOI:** 10.3897/zookeys.1141.84211

**Published:** 2023-01-18

**Authors:** Yan Zhi, Lin Yang, Xiang-Sheng Chen

**Affiliations:** 1 Key Laboratory of Medical Insects, Guizhou Medical University, Guiyang, Guizhou, 550025, China; 2 Institute of Entomology, Guizhou University, Guiyang, Guizhou, 550025, China; 3 The Provincial Special Key Laboratory for Development and Utilization of Insect Resources of Guizhou, Guizhou University, Guiyang, Guizhou, 550025, China

**Keywords:** Auchenorrhyncha, Eastern Palaearctic region, Oriental region, planthopper, Sino-Japanese region, taxonomy

## Abstract

Two new species of genus *Kuvera* Distant, 1906, *Kuveracampylotropa* Zhi & Chen, **sp. nov.** and *K.elongata* Zhi & Chen, **sp. nov.**, and a new Chinese record, *K.basarukini* Emeljanov, 1998, are described and illustrated from China. The females of two other species of *Kuvera*, *K.laticeps* (Metcalf, 1936) and *K.ussuriensis* (Vilbaste, 1968), are described for the first time. An updated identification key to Chinese species of *Kuvera* is given.

## ﻿Introduction

The planthopper genus *Kuvera* was established by Distant (1906), with *K.semihyalina* Distant, 1906 as the type species by original designation. This genus belongs to the tribe Semonini of subfamily Cixiinae (Hemiptera: Cixiidae). Diagnostic features of Semonini include that the postclypeus is swollen, its clypeofrontal suture is convex, and the median carina of frons is incomplete or obscure (Holzinger et al. 2002; Emeljanov 2002). Previously 25 species in this genus have been recorded successively from Eastern Palearctic, Sino-Japanese and Oriental regions (e.g., Distant 1906; Matsumura 1914; Muir 1922; Dlabola 1957; Vilbaste 1968; Anufriev 1987; Emeljanov 1998; Tsaur et al. 1991; Rahman et al. 2017; Luo et al. 2019; Bourgoin 2022). The latest taxonomic works on *Kuvera* by Luo et al. (2019) included a description of two new species from China, a checklist of species and an identification key to 13 Chinese species, which were useful additions to the knowledge of the Chinese fauna.

The present study of Chinese specimens has found two new species and a new Chinese record. Females of two species, *K.laticeps* (Metcalf, 1936) and *K.ussuriensis* (Vilbaste, 1968), are also described for the first time.

## ﻿Materials and methods

Morphological terminology follows Bourgoin (1987) for male genitalia, Bourgoin et al. (2015) for wing venation and Bourgoin (1993) for female genitalia. Body length was measured from apex of vertex to tip of forewing; vertex length represents the median length of the vertex (from the apical transverse carina to the tip of basal emargination). Fuchsin staining was used to highlight female genitalia structures studied. External morphology and drawings were done with the aid of a Leica MZ 12.5 stereomicroscope. Photographs were taken with KEYENCE VHX-6000 system. Illustrations were scanned with a CanoScan LiDE 200 and imported into Adobe Photoshop 7.0 for labeling and plate composition. The dissected male and female genitalia are preserved in glycerin in small plastic tubes pinned together with the specimens. Zoogeographic regionalization scheme follows Holt et al. (2013). The distribution map was prepared with SimpleMappr (Shorthouse 2010).

The type specimens are deposited in the Institute of Entomology, Guizhou University, Guiyang, Guizhou Province, China (**GUGC**).

## ﻿Taxonomy

### ﻿Genus Kuvera

Taxon classificationAnimaliaHemipteraCixiidae

Distant, 1906

288DE046-6B88-595F-9875-B9CD84192A54

Kuvera Distant, 1906: 261; Tsaur et al. 1991: 50; Anufriev and Emeljanov 1988: 443; Emeljanov 1998: 133; Luo et al. 2019: 46.
Latoliarus Dlabola, 1957: 271: synonymized by Emeljanov 1998: 133.

#### Type species.

*Kuverasemihyalina* Distant, 1906, original designation.

#### Diagnosis.

For the diagnosis of *Kuvera* see Luo et al. (2019: 137).

#### Remarks.

This genus is easily separated from other members in Semonini by the following character combinations: head including eyes narrower than pronotum; vertex short, wider than long, anterior margin of vertex obscure, with only residual traces; vertex narrowest at subapical carina, widening towards anterior and posterior margins; anterior and posterior margins wide and parabolic, almost parallel; frons prominent, median carina only distinct on basal portion, not reaching the anterior margin of vertex; clypeus swollen, postclypeus with prominent median carina, anteclypeus carina sharp or arcuate; rostrum just reaching hind coxae; forewings with ScP+R usually forked distad of CuA, RP 3-branched, MP with 4 or 5 terminals, CuA 2 or 3-branched, and with 10–11 apical cells; metatibiotarsal formula: 6/7/(7–8); pygofer with a triangular medioventral process; aedeagus with 2 spinose processes arising near base of endosoma, and endosoma with 1–2 spinose processes; periandrium almost flat and widened at base; ovipositor elongate, orthopteroid and apically curved upwards; abdominal 9^th^ tergite with a distinct and elliptic wax plate.

#### Distribution.

China, Korea, Japan, (Eastern) Russia, India, Myanmar, Afghanistan.

### ﻿Key to the known species (males) of *Kuvera* from China (revised from Anufriev 1987 and Luo et al. 2019)

**Table:** 

1	Forewing crossed before middle by a curved, slightly broken macular fuscous fascia (Distant 1906: fig. 117)	***K.semihyalina* Distant, 1906**
–	Forewing without fascia before middle	**2**
2	Pronotum white	***K.longipennis* Matsumura, 1914**
–	Pronotum yellow to dark brown	**3**
3	Spinose process of endosoma long, beyond the apex of the endosoma (Fig. 5H–J)	***K.elongata* sp. nov.**
–	Spinose process of endosoma not beyond the apex of the endosoma	**4**
4	One or both of the spinous processes on lateral sides of the periandrium curved to the opposite side over its dorsal surface	**5**
–	Neither of the spinous processes on lateral sides of the periandrium curved to the opposite side over its dorsal surface	**13**
5	Both spinose processes on lateral sides of the periandrium curved to the opposite side	**6**
–	Only one of the two spinose processes on lateral sides of the periandrium curved to the opposite side	**7**
6	Spinose process on right side of periandrium strongly curved, apex directed left-ventrocaudally (Anufriev 1987: figs 69, 70)	***K.toroensis* Matsumura, 1914**
–	Spinose process on right side of periandrium slightly curved, apex directed left-dorsocephalically (Tsaur et al. 1991: fig. 28)	***K.transversa* Tsaur & Hsu, 1991**
7	Spinose process on right side of periandrium curved to left side (Fig. 6H–K)	***K.laticeps* (Metcalf, 1936)**
–	Spinose process on left side of periandrium curved to right side	**8**
8	Left spinose process of periandrium S-shaped	**9**
–	Left spinose process of periandrium not S-shaped	**11**
9	Anal segment with apical lobes symmetrical (Tsaur et al. 1991: fig. 30)	***K.hama* Tsaur & Hsu, 1991**
–	Anal segment with apical lobes asymmetrical	**10**
10	Apex of left process reaching base of periandrium; endosoma process reaching apex of sclerotized portion of endosoma (Luo et al. 2019: figs 10, 22)	***K.huoditangensis* Luo, Liu & Feng, 2019**
–	Apex of left process not reaching base of periandrium; endosoma process reaching middle of membranous portion of endosoma (Anufriev 1987: figs 20–22)	***K.vilbastei* Anufriev, 1987**
11	Endosoma process long, longer than two-thirds of the left spinose process of periandrium (Luo et al. 2019: figs 43, 44)	***K.longwangshanensis* Luo, Liu & Feng, 2019**
–	Endosoma process short, shorter than half of the left spinose process of periandrium	**12**
12	Anal tube more or less parallel-sided in dorsal view; apex of left spinose process of periandrium exceeding right lateral margin of periandrium (Fig. 3 F, J, K)	***K.basarukini* Emeljanov, 1998**
–	Anal tube widened in the middle in dorsal view; apex of left spinose process of periandrium not reaching right lateral margin of periandrium (Anufriev 1987: figs 42–45)	***K.flaviceps* (Matsumura, 1900)**
13	Right process of periandrium originated ventral surface	**14**
–	Right process of periandrium originated right side	**15**
14	Anal segment with apical lobes symmetrical (Anufriev 1987: fig. 67)	***K.ussuriensis* (Vilbaste, 1968)**
–	Anal segment with apical lobes asymmetrical (Rahman et al. 2017; Fig. 6F)	***K.yecheonensis* Rahman, Kwon & Suh, 2017**
15	The two spinous processes of periandrium nearly equal in length	**16**
–	The two spinous processes of periandrium not of equal length	**18**
16	In lateral view, two spinose processes of periandrium arched (Tsaur et al. 1991: fig. 25)	***K.taiwana* Tsaur & Hsu, 1991**
–	In lateral view, two spinose processes of periandrium almost straight	**17**
17	In dorsal view, two spinose processes of periandrium curved inwards (Anufriev 1987: fig. 50)	***K.kurilensis* Anufriev, 1987**
–	In dorsal view, two spinose processes of periandrium almost straight (Anufriev 1987: fig. 57)	***K.tappanella* Matsumura, 1914**
18	Right process of periandrium longer than left one (Fig. 4H–K)	***K.campylotropa* sp. nov.**
–	Left process of periandrium longer than right one	**19**
19	Ventral base of periandrium triangular (Tsaur et al. 1991: fig. 29)	***K.communis* Tsaur & Hsu, 1991**
–	Ventral base of periandrium roundly concaved (Tsaur et al. 1991: fig. 27)	***K.similis* Tsaur & Hsu, 1991**

### ﻿ Kuvera basarukini

Taxon classificationAnimaliaHemipteraCixiidae

Emeljanov, 1998

16980AFC-2A91-571D-B5D6-9D20CB766FDF

Figs 1A, B , 3

Kuvera basarukini Emeljanov, 1998: 133.

#### Material examined.

**China**: 1♂, Guizhou Province, Duyun City, Doupengshan (26°21'N, 107°23'E), 19 August 2017, leg. Liang-Jing Yang; 1♂, Guizhou Province, Rongjiang county, Xiaodanjiang (660–800 m) (26°20'N, 108°21'E), 13–14 September 2005, leg. Bin Zhang, Zi-Zhong Li.

#### Redescription.

Body length: male: 5.5–5.9 mm (*N* = 2).

**Figure 1.**
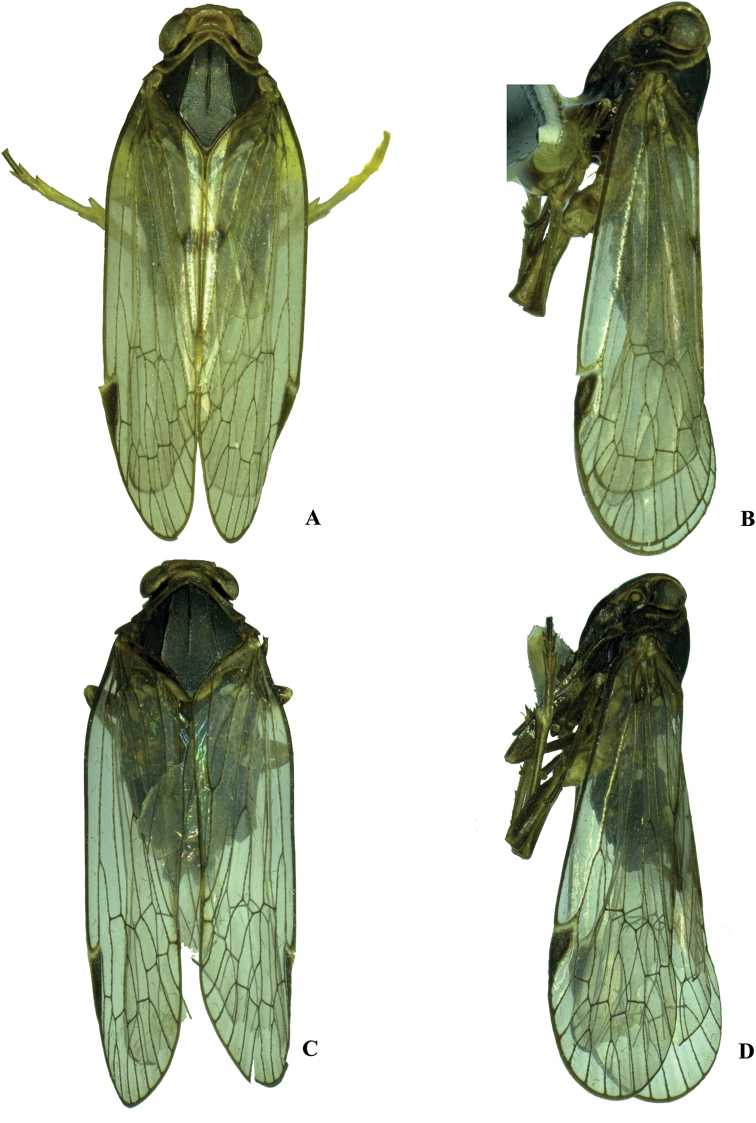
Habitus **A, B***Kuverabasarukini* Emeljanov, 1998, male **A** dorsal view **B** lateral view **C, D***Kuveracampylotropa* sp. nov., male **C** dorsal view **D** lateral view.

***Coloration*.** General color blackish brown (Figs 1A, B, 3A, B). Eyes brown, ocelli yellowish brown. Vertex brown, pronotum dark brown and mesonotum blackish brown. Frons generally yellowish brown, blackish brown above frontoclypeal suture. Clypeus blackish brown. Rostrum generally brown except darker tip. Forewing semi-translucent, with a small irregular blackish brown spot at branch of Y-vein, stigma blackish brown. Hind tibiae yellowish brown and abdominal sternites blackish brown.

**Figure 2.**
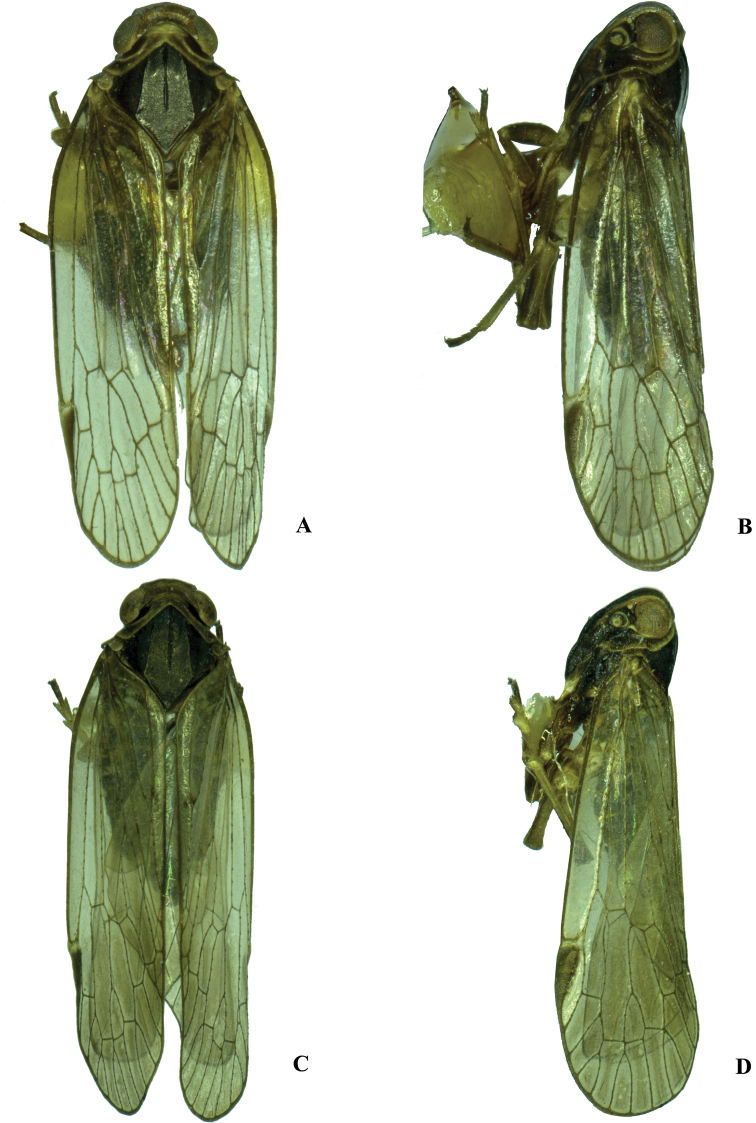
Habitus **A, B***Kuveraelongata* sp. nov., male **A** dorsal view **B** lateral view **C, D***Kuveralaticeps* (Metcalf, 1936), male **C** dorsal view **D** lateral view.

**Figure 3.**
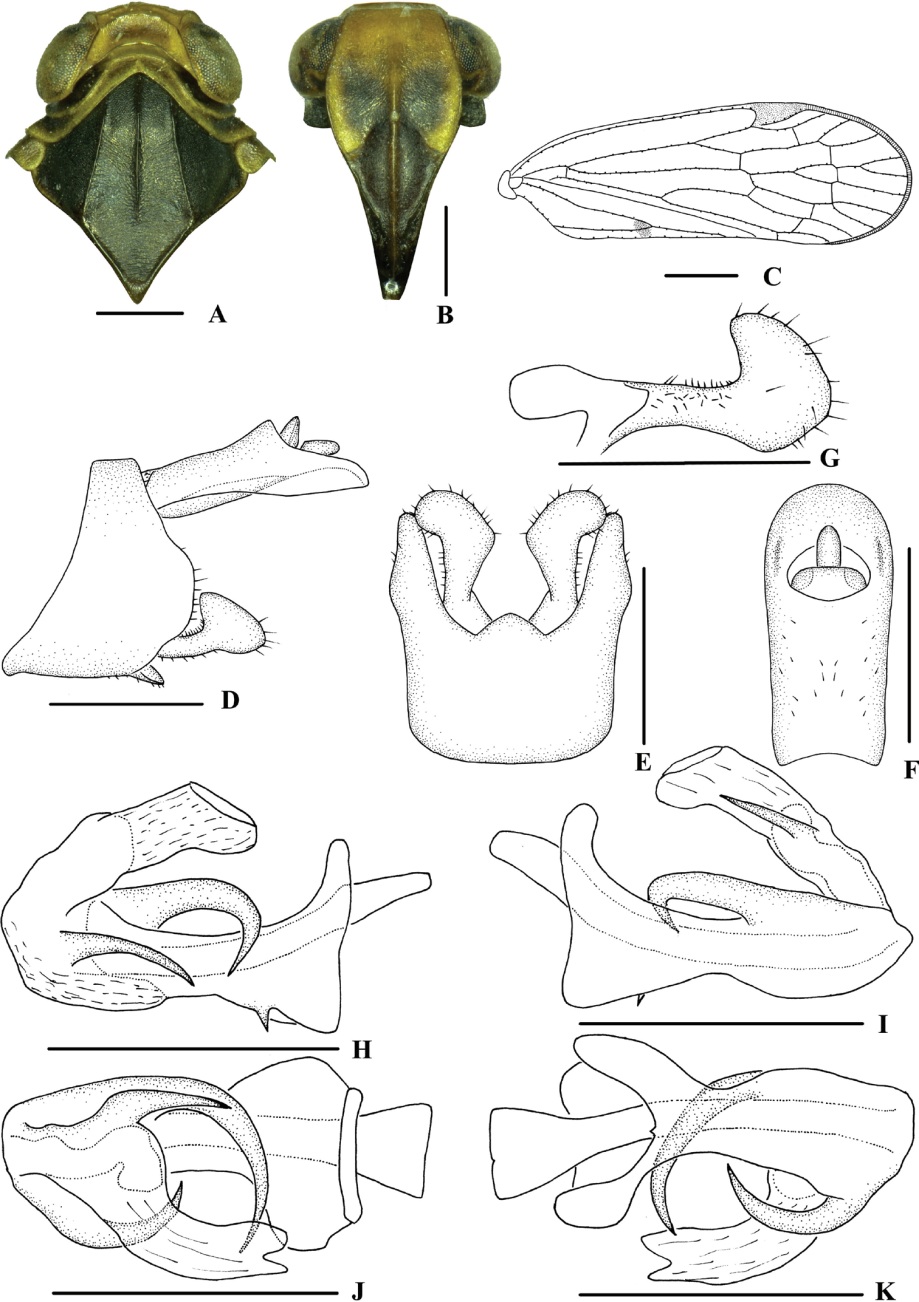
*Kuverabasarukini* Emeljanov, 1998, male **A** head and thorax, dorsal view **B** face, ventral view **C** forewing **D** genitalia, lateral view **E** pygofer and gonostyli, ventral view **F** anal segment, dorsal view **G** gonostyli, inner lateral view **H** aedeagus, right side **I** aedeagus, left side **J** aedeagus, dorsal view **K** aedeagus, ventral view. Scale bars: 0.5 mm (**A, B, D–K**); 1.0 mm (**C**).

***Head and thorax*.** Vertex (Fig. 3A) broad, 2.2 times wider than long; anterior margin arched convex, posterior margin arched concave; median carina reaching transverse carinae, indistinct. Frons (Fig. 3B) 1.2 times as wide as long, median carina indistinct, extending from slightly above level of lateral ocelli to median ocellus. Clypeus with median carina distinct and elevated throughout. Pronotum (Fig. 3A) 2.2 times longer than vertex, posterior margin nearly at right angle. Mesonotum 1.6 times longer than pronotum and vertex combined. Forewing (Fig. 3C) 3.0 times longer than wide, with 10 apical and 6 subapical cells; fork Sc+RP distad of fork CuA_1_+CuA_2_; first crossvein r-m basad of fork MP; RP 2 branches, MP with five terminals: MP_11_, MP_12_, MP_2_, MP_3_, and MP_4_, fork MP_1_+MP_2_ basad of fork MP_3_+MP_4_. Hind tibia with 2–3 lateral spines, metatibiotarsal formula: 6/7/7–8, second segment of hind tarsus with 2–3 platellae.

***Male genitalia*.** Pygofer (Fig. 3D, E) symmetrical, dorsal margin concave and U-shaped ventrally, widened towards apex; in lateral view, lateral lobes arched extended caudally. Medioventral process triangular in ventral view. Anal segment (Fig. 3D, F) long, tubular, symmetrical, apical lobes slightly enlarged, 2.2 times longer than wide in dorsal view; anal style finger-like, not extending beyond anal segment. Gonostyli (Fig. 3D, E, G) symmetrical in ventral view; in inner lateral view, apical part extended, apical margin round. Aedeagus (Fig. 3H–K) in total with three processes. Right apex of periandrium with a medium-sized spinose process, curved and apex directed left-ventrocephalad; spinose process on left side near apical 1/3 of periandrium being the longest, gently curved from left to right side over periandrium and apex directed to the right side; base of periandrium ventrally with one small tooth. Endosoma slender, structure simple, left side near the middle with a stout and short spinose process, apex directed dorsocephalad.

#### Host plant.

Unknown.

#### Distribution.

China (Guizhou), Russia (Sakhalin Island).

#### Remarks.

This species can be distinguished from other species of the genus by the following characters: anal segment symmetrical; aedeagus with three processes: right spinose process of periandrium curved and apex directed left-ventrocephalad; left spinose process of periandrium being the longest, curved over periandrium and apex exceeding right lateral margin of periandrium; spinose process of endosoma stout and short, apex directed dorsocephalad.

#### Note.

This species is recorded from China for the first time.

### ﻿ Kuvera campylotropa

Taxon classificationAnimaliaHemipteraCixiidae

Zhi & Chen sp. nov.

8D8FA8B9-BFB6-5C16-9735-CB062710185F

https://zoobank.org/6F94F366-14A5-4582-ABDD-5CBA315DDAEF

Figs 1C, D , 4

#### Type material.

***Holotype***: ♂, **China**: Yunnan Province, Lushui City, Pianma Town (26°1'N, 98°37'E), 17 June 2011, leg. Yu-Jian Li, Jian-Kun Long; paratypes: 1♂ 1♀, same data as holotype; 6♂♂ 1♀, Guizhou Province, Daozhen County, Xiannvdong Nature Reserve (29°3'N, 107°25'E), 26 August 2004, leg. Xiang-Sheng Chen.

#### Description.

Body length: male 5.1–6.3 mm (*N* = 8), female 6.1–6.5 mm (*N* = 2).

**Figure 4.**
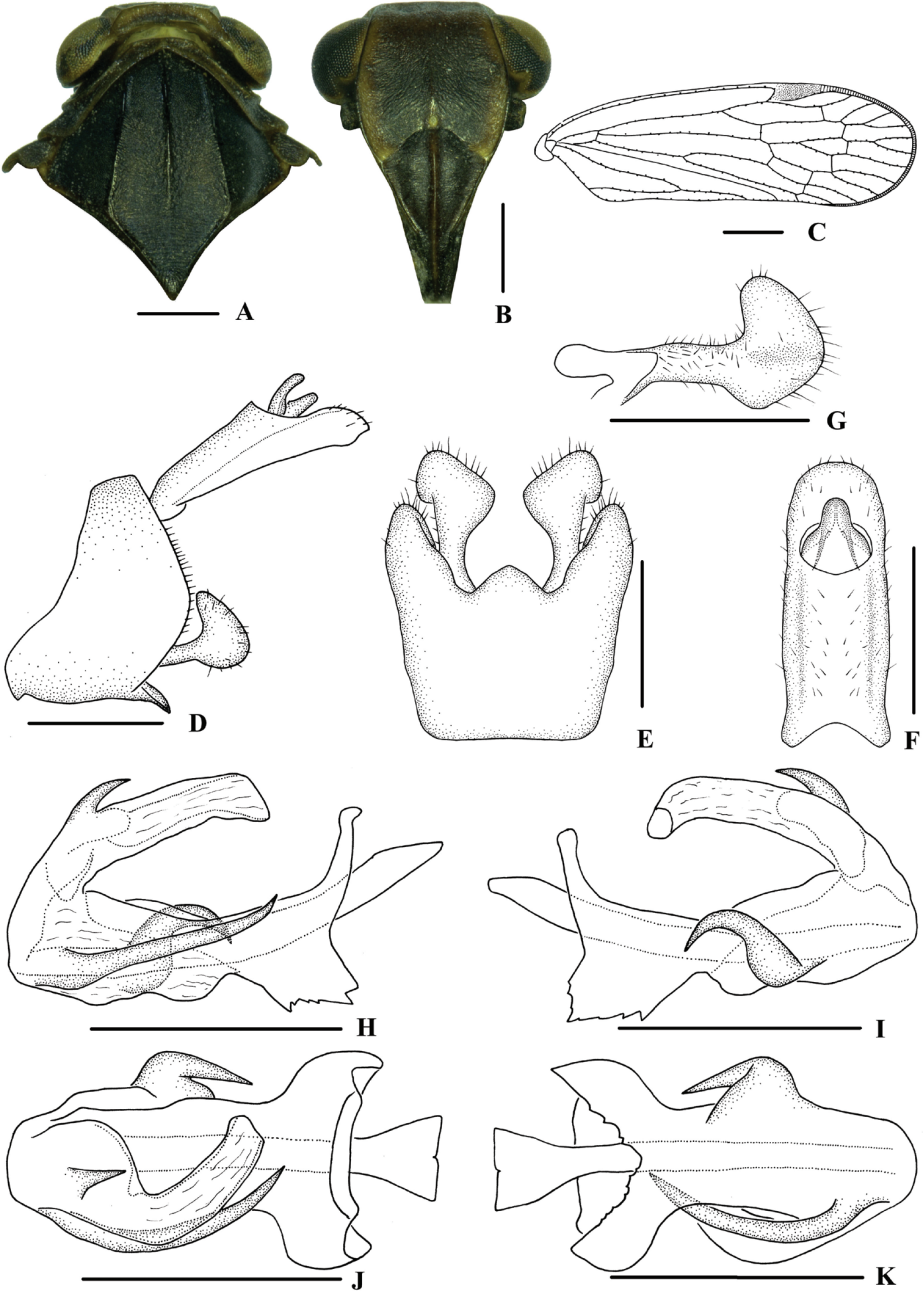
*Kuveracampylotropa* sp. nov., male **A** head and thorax, dorsal view **B** face, ventral view **C** forewing **D** genitalia, lateral view **E** pygofer and gonostyli, ventral view **F** anal segment, dorsal view **G** gonostyli, inner lateral view **H** aedeagus, right side **I** aedeagus, left side **J** aedeagus, dorsal view **K** aedeagus, ventral view. Scale bars: 0.5 mm (**A, B, D–K**); 1.0 mm (**C**).

***Coloration*.** General color blackish brown (Figs 1C, D, 4A, B). Eyes dark brown, ocelli yellowish brown. Vertex dark brown, pronotum dark brown and mesonotum blackish brown. Face generally blackish brown. Rostrum generally dark brown except darker tip. Forewing semi-translucent, stigma blackish brown. Hind tibiae brown and abdominal sternites blackish brown.

***Head and thorax*.** Vertex (Fig. 4A) broad, 4.2 times wider than long; anterior margin slightly arched convex, posterior margin slightly arched concave; median carina reaching transverse carinae. Frons (Fig. 4B) 1.2 times as wide as long, median carina indistinct, extending from slightly above level of lateral ocelli to median ocellus. Clypeus with median carina distinct and elevated throughout. Pronotum (Fig. 4A) 4.4 times longer than vertex, posterior margin nearly at right angle. Mesonotum 1.9 times longer than pronotum and vertex combined. Forewing (Fig. 4C) 3.1 times longer than wide, with 11 apical and 6 subapical cells; fork Sc+RP distad of fork CuA_1_+CuA_2_; first crossvein r-m basad of fork MP; RP 3 branches, MP with five terminals: MP_11_, MP_12_, MP_2_, MP_3_, and MP_4_, fork MP_1_+MP_2_ basad of fork MP_3_+MP_4_. Hind tibia with 3 lateral spines, metatibiotarsal formula: 6/7/8, second segment of hind tarsus with 3 platellae.

***Male genitalia*.** Pygofer (Fig. 4D, E) symmetrical, dorsal margin concave and U-shaped ventrally, widened towards apex; in lateral view, lateral lobes arched extended caudally. Medioventral process triangular in ventral view. Anal segment (Fig. 4D, F) long, tubular, symmetrical, apical lobes slightly enlarged, 2.6 times longer than wide in dorsal view; anal style finger-like, not extending beyond anal segment. Gonostyli (Fig. 4D, E, G) symmetrical in ventral view; in inner lateral view, apical part extended, apical margin round. Aedeagus (Fig. 4H–K) in total with three processes. Spinose process on right side at apex of periandrium being the longest, slightly curved and apex directed left-dorsocephalad; left side in the middle with a strongly curved spinose process, apex directed ventrocephalad; base of periandrium ventrally with several small teeth. Endosoma slender, structure simple, dorsal margin near the middle with a stout and short spinose process, apex directed dorsocephalad.

#### Host plant.

Unknown.

#### Distribution.

China (Guizhou, Yunnan).

#### Etymology.

The specific name is derived from the Latin *campylotropus*, meaning curved, referring to the strong curved spinose process on the left side of periandrium.

#### Remarks.

This new species is similar to *K.ussuriensis* (Vilbaste, 1968), but differs in: (1) “right” spinose process of periandrium originating from right apex (in *K.ussuriensis*, “right” spinose process of periandrium originating from ventral apex); (2) left spinose process of periandrium shorter than right one in lateral view (the latter longer than right one); and (3) spinose process of endosoma not reaching apex of endosoma (in *K.ussuriensis*, spinose process of the endosoma nearly reaching apex of endosoma). It also closely resembles *Kuverakurilensis* Anufriev, 1987, however, it differs in that: (1) right spinose process of periandrium longer than left one in lateral view (in *K.kurilensis*, both processes about equal in length); and (2) spinose process of endosoma not reaching apex of endosoma (in *K.kurilensis*, spinose process of endosoma nearly reaching apex of endosoma).

### ﻿ Kuvera elongata

Taxon classificationAnimaliaHemipteraCixiidae

Zhi & Chen sp. nov.

96E8F8AB-A2D9-5E7B-B27E-D213310B47C9

https://zoobank.org/0BB30DF7-8F44-4486-B262-8E069F7892E8

Figs 2A, B , 5

#### Type material.

***Holotype***: ♂, **China**: Guizhou Province, Tongren City, Fanjingshan National Nature Reserve, Jinding (27°54'N, 108°42'E), 31 May 2002, leg. Xiang-Sheng Chen; ***paratypes***: 8♂♂ 1♀, same data as holotype; 2♂♂, Guizhou Province, Tongren City, Fanjingshan National Nature Reserve, Yinjiang County, Yongyi Township, (27°54'N, 108°38'E), 29 May 2002, leg. Xiang-Sheng Chen.

#### Description.

Body length: male 4.8–5.8 mm (*N* = 11), female 6.0 mm (*N* = 1).

**Figure 5.**
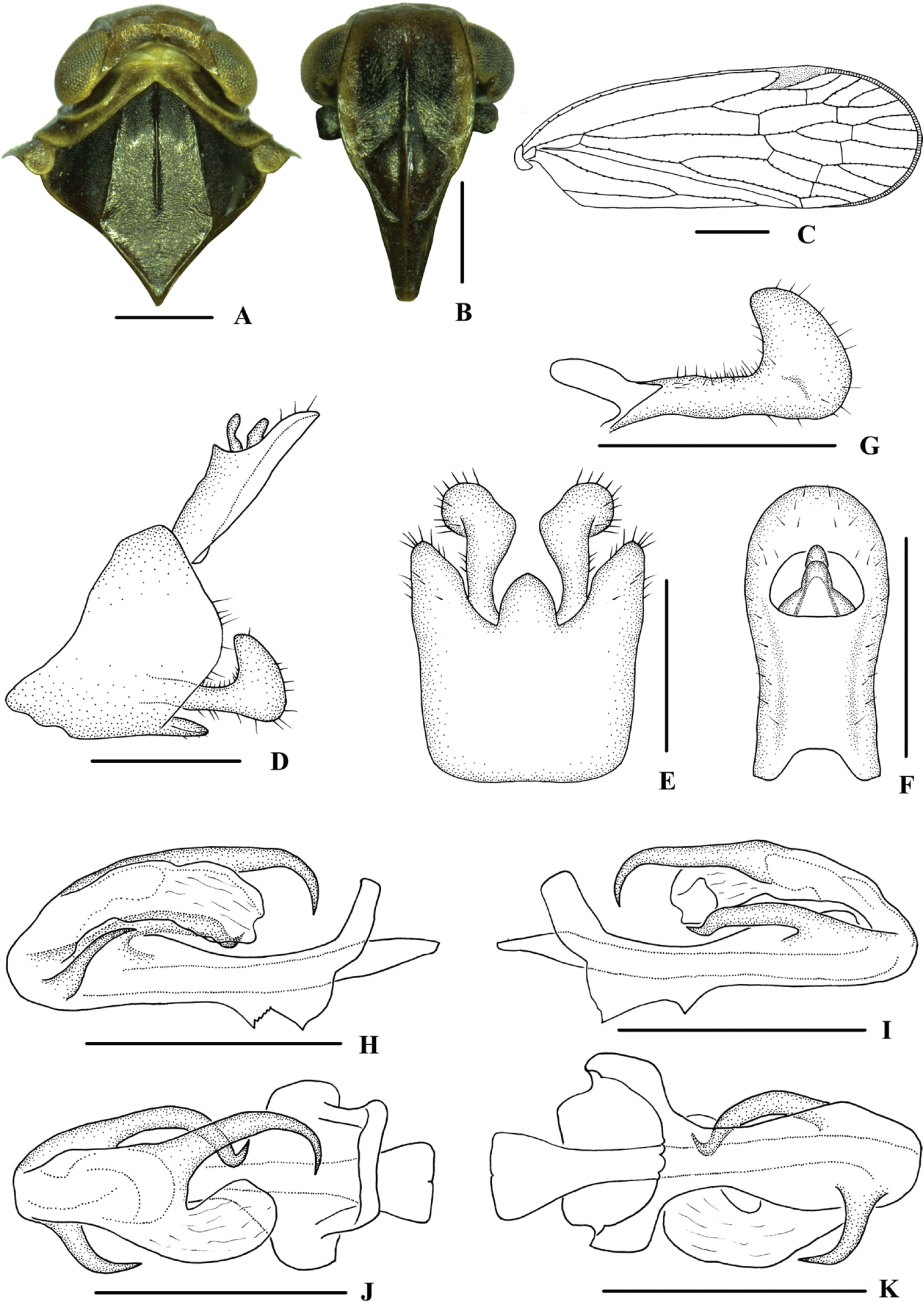
*Kuveraelongata* sp. nov., male **A** head and thorax, dorsal view **B** face, ventral view **C** forewing **D** genitalia, lateral view **E** pygofer and gonostyli, ventral view **F** anal segment, dorsal view **G** gonostyli, inner lateral view **H** aedeagus, right side **I** aedeagus, left side **J** aedeagus, dorsal view **K** aedeagus, ventral view. Scale bars: 0.5 mm (**A, B, D–K**); 1.0 mm (**C**).

***Coloration*.** General color blackish brown (Figs 2A, B, 5A, B). Eyes dark brown, ocelli light yellowish. Vertex brown, pronotum dark brown and mesonotum blackish brown. Face generally blackish brown. Rostrum generally brown except darker tip. Forewing semi-translucent, stigma dark brown. Hind tibiae brown and abdominal sternites blackish brown.

***Head and thorax*.** Vertex (Fig. 5A) broad, 2.7 times wider than long; anterior margin slightly arched convex, posterior margin arched concave; median carina reaching transverse carinae. Frons (Fig. 5B) 1.1 times as wide as long, median carina indistinct, extending from basal 1/4 to median ocellus. Clypeus with median carina distinct and elevated throughout. Pronotum (Fig. 5A) 2.3 times longer than vertex, posterior margin nearly at right angle. Mesonotum 1.6 times longer than pronotum and vertex combined. Forewing (Fig. 5C) 2.7 times longer than wide, with 11 apical and 6 subapical cells; fork Sc+RP distad of fork CuA_1_+CuA_2_; first crossvein r-m basad of fork MP; RP 3 branches, MP with five terminals: MP_11_, MP_12_, MP_2_, MP_3_, and MP_4_, fork MP_1_+MP_2_ basad of fork MP_3_+MP_4_. Hind tibia with 3 lateral spines, metatibiotarsal formula: 6/7/8, second segment of hind tarsus with 2–3 platellae.

***Male genitalia*.** Pygofer (Fig. 5D, E) symmetrical, dorsal margin concave and U-shaped ventrally, widened towards apex; in lateral view, lateral lobes arched extended caudally. Medioventral process campanulate in ventral view. Anal segment (Fig. 5D, F) long, tubular, symmetrical, apical lobes slightly enlarged, 2.0 times longer than wide in dorsal view; anal style finger-like, not extending beyond anal segment. Gonostyli (Fig. 5D, E, G) symmetrical in ventral view; in inner lateral view, apical part extended, apical margin round. Aedeagus (Fig. 5H–K) with three processes in total. Spinose process on right side at apex of periandrium being the shortest, slightly curved outward and apex directed dorsocephalad; left side near base with a slightly curved long spinose process, apex strongly recurved and directed to left side; base of periandrium ventrally with several small teeth. Endosoma slender, structure simple, left side near the middle with a stout and long spinose process, which extended beyond the apex of the endosoma, apex directed ventrad.

#### Host plant.

Grass.

#### Distribution.

China (Guizhou).

#### Etymology.

The specific name is derived from the Latin *elongatus*, meaning elongated, referring to the elongated spinose process on the left side of endosoma.

#### Remarks.

This new species is similar to *K.vilbastei* Anufriev, 1987 and *K.huoditangensis* Luo, Liu & Feng, 2019, but differs in: (1) left spinose process of periandrium not exceeding right lateral margin of periandrium (in *K.vilbastei* and *K.huoditangensis*, left spinose process of periandrium exceeding right lateral margin of periandrium); (2) spinose process of endosoma extending beyond the apex of the endosoma (spinose process of endosoma not extending beyond the apex of the endosoma in *K.vilbastei* and *K.huoditangensis*); and (3) anal segment symmetrical (asymmetrical in *K.vilbastei* and *K.huoditangensis*).

### ﻿ Kuvera laticeps

Taxon classificationAnimaliaHemipteraCixiidae

(Metcalf, 1936)

C377AFEF-B529-5926-9072-66416E54E518

Figs 2C, D , 6 , 7

Cixius latifrons Melichar, 1902: 85, preoccupied by Cixiuslatifrons Walker, 1851.
Cixius laticeps Metcalf, 1936: 180, nom. nov. for Cixiuslatifrons Melichar, 1902.
Kuvera laticeps (Metcalf, 1936): combination by Anufriev 1987: 6.

#### Material examined.

**China**: 16♂♂ 22♀♀, Guizhou Province, Weining County, Xueshan Town (27°11'N, 104°6'E), 28–29 September 2016, leg. Jian-Kun Long, Hong-Xing Li, Ya-Lin Yao.

#### Description.

Body length: male 5.4–6.2 mm (*N* = 16), female 6.1–6.8 mm (*N* = 22).

**Figure 6.**
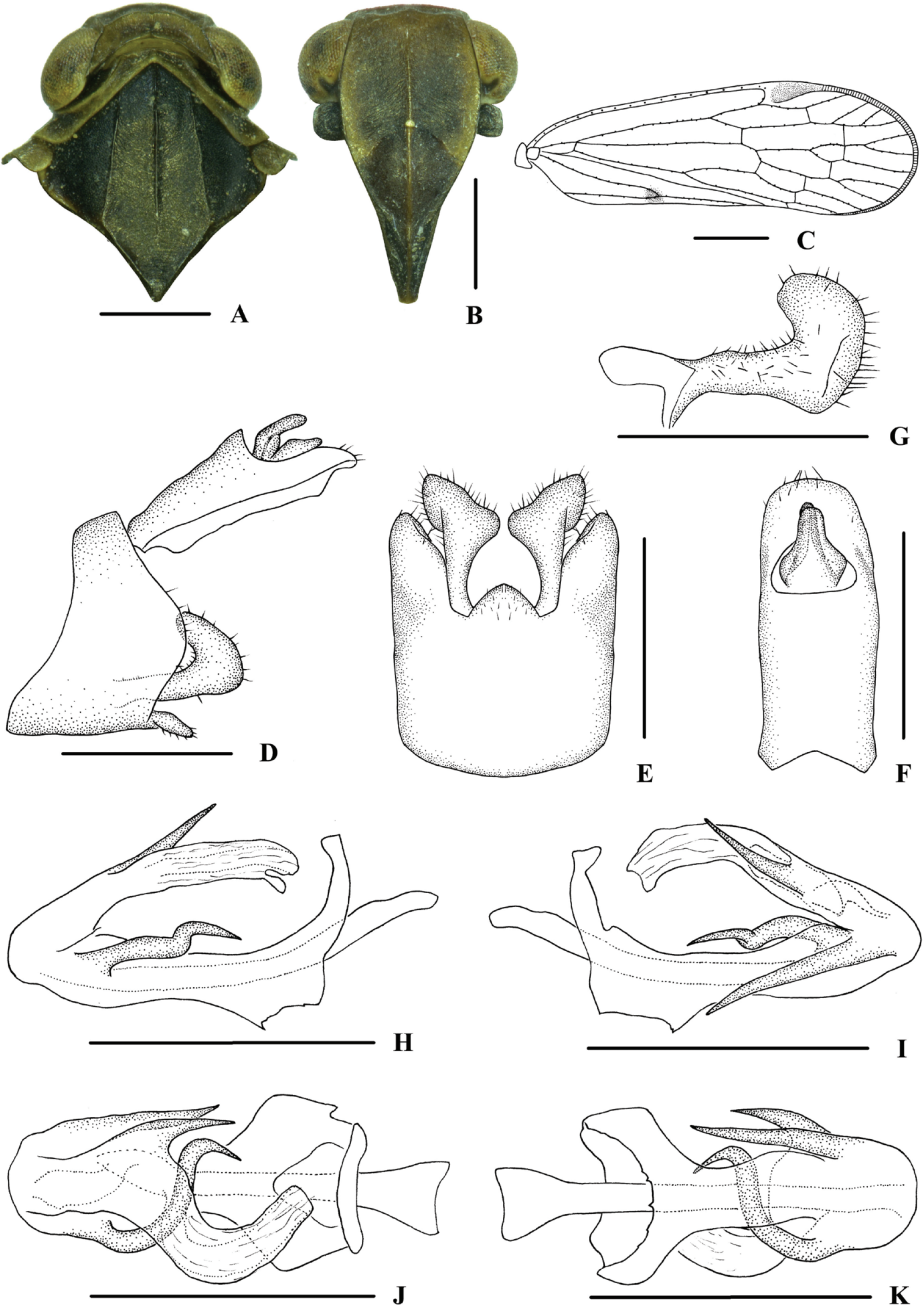
*Kuveralaticeps* (Metcalf, 1936), male **A** head and thorax, dorsal view **B** face, ventral view **C** forewing **D** genitalia, lateral view **E** pygofer and gonostyli, ventral view **F** anal segment, dorsal view **G** gonostyli, inner lateral view **H** aedeagus, right side **I** aedeagus, left side **J** aedeagus, dorsal view **K** aedeagus, ventral view. Scale bars: 0.5 mm (**A, B, D–K**); 1.0 mm (**C**).

***Coloration*.** General color blackish brown (Figs 2C, D, 6A, B). Eyes brown, ocelli yellow. Vertex brown, pronotum brown and mesonotum blackish brown. Frons generally brown and clypeus blackish brown. Rostrum generally brown except darker tip. Forewing semi-translucent, with a very small irregular blackish brown spot at branch of Y-vein, stigma dark brown. Hind tibiae brown and abdominal sternites blackish brown.

***Head and thorax*.** Vertex (Fig. 6A) broad, 3.0 times wider than long; anterior margin slightly arched convex, posterior margin arched concave; median carina reaching transverse carinae. Frons (Fig. 6B) 1.2 times as wide as long, median carina indistinct, extending from basal 1/4 to median ocellus. Clypeus with median carina distinct and elevated throughout. Pronotum (Fig. 6A) 2.8 times longer than vertex, posterior margin nearly at right angle. Mesonotum 1.6 times longer than pronotum and vertex combined. Forewing (Fig. 6C) 3.0 times longer than wide, with 11 apical and 6 subapical cells; fork Sc+RP distad of fork CuA_1_+CuA_2_; first crossvein r-m basad of fork MP; RP 3 branches, MP with five terminals: MP_11_, MP_12_, MP_2_, MP_3_, and MP_4_, fork MP_1_+MP_2_ basad of fork MP_3_+MP_4_. Hind tibia with 3–4 lateral spines, metatibiotarsal formula: 6/7/8, second segment of hind tarsus with 2–4 platellae.

***Male genitalia*.** Pygofer (Fig. 6D, E) symmetrical, dorsal margin concave and U-shaped ventrally, slightly widened towards apex; in lateral view, lateral lobes arched extended caudally. Medioventral process triangular in ventral view. Anal segment (Fig. 6D, F) long, tubular, nearly symmetrical, apical lobes slightly enlarged, 2.4 times longer than wide in dorsal view; anal style finger-like, not extending beyond anal segment. Gonostyli (Fig. 6D, E, G) symmetrical in ventral view; in inner lateral view, apical part extended, apical margin round. Aedeagus (Fig. 6H–K) in total with three processes. Spinose process on right side at apex of periandrium being the longest, gently curved from right to left side over periandrium, apex strongly recurved at 90° and directed apically; left side near base with a straight long spinose process, apex directed ventrocephalad; base of periandrium ventrally with several very small teeth. Endosoma slender, structure simple, left side near the middle with a stout and short spinose process, nearly straight, apex directed dorsocephalad.

**Figure 7.**
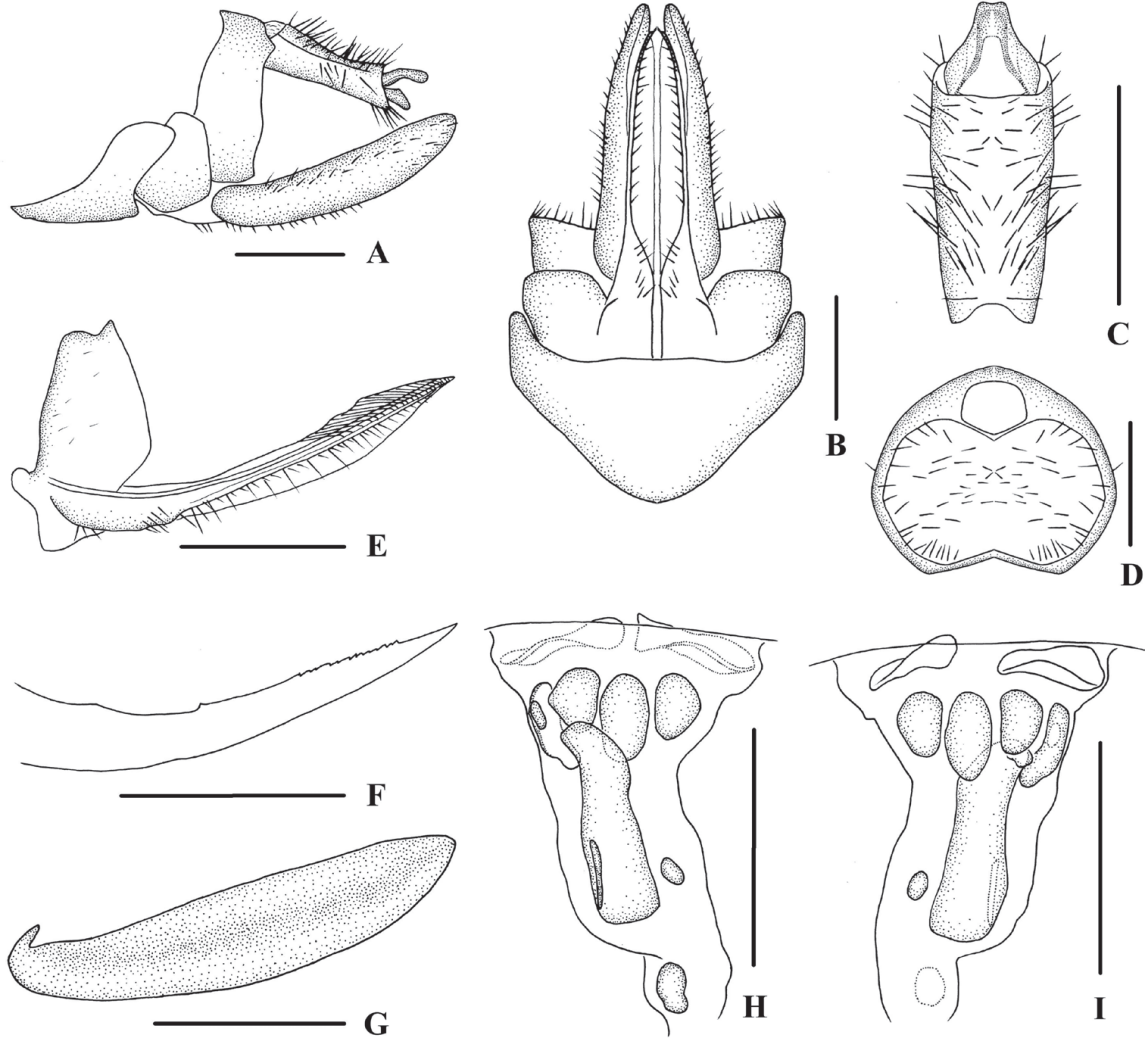
*Kuveralaticeps* (Metcalf, 1936), female **A** genitalia, lateral view **B** genitalia, ventral view **C** anal segment, dorsal view **D** tergite IX, caudal view **E** gonapophysis VIII and gonocoxa VIII, ventral view **F** gonapophysis IX, lateral view **G** gonoplac, inner lateral view **H** posterior vagina, ventral view **I** posterior vagina, dorsal view. Scale bars: 0.5 mm.

***Female genitalia*.** Tergite IX (Fig. 7A, B, D) moderately sclerotized, with a large wax plate, nearly oval, dorsal and ventral margins concave. Anal segment (Fig. 7C) rectangular, 2.1 times wider than long in dorsal view, anal style strap-like. Gonapophysis VIII (Fig. 7E) elongate, and slightly curved upwards. Gonapophysis IX (Fig. 7F) with two middle teeth, distance ratio between distal middle tooth to apex and length of denticulate portion is 2.6. Gonoplac (Fig. 7G) rod-like, 4.6 times longer than wide in lateral view. Posterior vagina pattern as shown in Figure 7H, I.

#### Host plant.

Unknown.

#### Distribution.

China (Guizhou, Sichuan).

#### Note.

The female genitalia of this species are described and illustrated for the first time.

#### Remarks.

This species can be distinguished from other species of the genus by the following characters: anal segment symmetrical; right spinose process of periandrium being the longest, S-shaped, curved over periandrium and apex exceeding left lateral margin of periandrium; left spinose process of periandrium straight, apex directed ventrocephalad; spinose process of endosoma stout and short straight, apex directed dorsocephalad.

### ﻿ Kuvera ussuriensis

Taxon classificationAnimaliaHemipteraCixiidae

(Vilbaste, 1968)

5A09DA90-F125-5785-9A95-0DA0C6DAF648

Figs 8 , 9 , 10

Betacixius ussuriensis Vilbaste, 1968: 9.
Kuvera ussuriensis (Vilbaste, 1968): combination by Anufriev 1987: 17.

#### Material examined.

**China**: 4♂♂ 7♀♀, Hebei Province, Xinglong County, Wulingshan National Nature Reserve (40°36'N, 117°29'E), 14 August 2010, leg. Li-Xia Xie, Da-Xing Yang, Rong Huang; 50♂♂ 38♀♀, Shanxi Province, Yicheng County, Lishan National Nature Reserve, Dahe Forest Farm (35°27'N, 111°56'E), 23–25 July 2012, leg. Pei Zhang; 28♂♂ 23♀♀, Shanxi Province, Qinshui County, Zhongcun Town, Zhangma Village (35°35'N, 111°57'E), 22 July 2012, leg. Pei Zhang; 26♂♂ 28♀♀, Shanxi Province, Lishan National Nature Reserve (35°23'N, 111°59'E) (1300–2200 m), 31 July 2012, leg. Pei Zhang; 31♂♂ 23♀♀, Shanxi Province, Lishan National Nature Reserve (1300–2200 m), 12–18 July 2012, leg. Xiao-Hui Hou; 1♂ 4♀♀, Sichuan Province, Nanchong City, Dayou Township (30°48'N, 106°41'E), 10 May 2008, leg. Zai-Hua Yang; 5♂♂ 3♀♀, Sichuan Province, Luding County, Yanzigou Town (29°42'N, 102°1'E), 11 August 2015, leg. Hong-Ping Zhan, Wen-Song Li; 2♂♂ 2♀♀, Sichuan Province, Qingchuan County, Tangjiahe National Nature Reserve (32°35'N, 104°46'E), 24 August 2007, leg. Ze-Hong Meng; 6♂♂ 10♀♀, Mianyang City, Pingwu County, Baima Tibetan Township, Wanglang Nature Reserve (32°54'N, 104°9'E); leg. Zai-Hua Yang, Wen Zhang; 2♂♂ 4♀♀, Yunnan Province, Yingjiang County, Xima Town (24°45'N, 97°42'E), 29–30 May 2011, leg. Zai-Hua Yang, Jian-Kun Long; 4♂♂, Yunnan Province, Pingbian County, Daweishan National Nature Reserve (22°56'N, 103°42'E), 20 August 2017, leg. Nian Gong; 10♂♂ 13♀♀, Yunnan Province, Xichou County, Fadou (23°23'N, 104°47'E), 28 June 2013, leg. Yan Zhi, Qiang Luo, Yong-Jin Sui; 2♂♂ 2♀♀, Yunnan Province, Maguan County, Dulong Town, Jinzhuping Village (22°56'N, 104°30'E), 13–14 August 2017, leg. Yan Zhi, Qiang Luo, Nian Gong; 8♂♂ 8♀♀, Guangxi Province, Longsheng County, Huaping National Nature Reserve (25°36'N, 109°56'E), 26 April 2012, leg. Jian-Kun Long, Zai-Hua Yang; 7♂♂ 3♀♀, Guangxi Province, Longsheng County, Huaping National Nature Reserve, 18–19 May 2012, leg. Jian-Kun Long, Zhi-Hua Fan; 9♂♂ 10♀♀, Shaanxi Province, Zhouzhi County, Houzhenzi Town (33°51'N, 107°50'E), 4–7 August 2010, leg. Pei Zhang, Zhi-Min Chang, Yan-Li Zheng, Ke-Bin Li; 5♂♂ 5♀♀, Shaanxi Province Xi’an City, Cuihuashan (33°58'N, 109°1'E), 27–28 August 2008, leg. Yu-Jian Li; 2♂♂ 2♀♀, Shaanxi Province, Taibai County (34°4'N, 107°19'E), 22 August 2016, leg. Nian Gong; 2♂♂ 5♀♀, Hunan Province, Wugang City, Yunshan National Forest Park (26°40'N, 110°37'E), May 2016, leg. Xiang-Sheng Chen; 2♂♂, Hunan Province, Yongshun County, Xiaoxi Town (28°44'N, 110°15'E), 20–21 August 2016, leg. Yong-Shun Ding, Ying-Jian Wang; 6♂♂ 2♀♀, Anhui Province, Huangshan city, Tangkou town (30°4'N, 118°11'E) (500m), 20 May 2008, leg. Zheng-Guang Zhang; 22♂♂ 33♀♀, Guizhou Province, Weining County, Caohai National Nature Reserve (26°52'N, 104°14'E) (2171 m), 1–5 August 2017, leg. Caohai Collection Team; 1♂ 5♀♀, Guizhou Province, Weining County, Xueshan Town, Zhuopu Village (27°11'N, 104°6'E), 21 August 1986, leg. Zi-Zhong Li; 6♂♂ 17♀♀, Guizhou Province, Daozhen County, Xiannvdong (29°3'N, 107°25'E), 29–31 May 2004, leg. Bin Zhang, Pian Xu; 25♂♂ 44♀♀, Guizhou Province, Daozhen County, Sanqiao Town (29°3'N, 107°30'E), 22–24 May 2004, leg. Xiang-Sheng Chen, Bin Zhang, Pian Xu; 4♂♂ 17♀♀, Guizhou Province, Daozhen County, Dashahe Nature Reserve (29°9'N, 107°36'E), 29–31 May 2004, leg. Xiang-Sheng Chen; 2♂♂ 3♀♀, Guizhou Province, Daozhen County, Dashahe Nature Reserve, 20 August 2004, leg. Xiang-Sheng Chen; 3♂♂, Guizhou Province, Luodian County, Luosha Township, Zheren Village (25°41'N, 106°36'E), 9 May 2013, leg. Jian-Kun Long; 6♂♂ 14♀♀, Guizhou Province, Anlong County (25°5'N, 105°29'E), 27 August 2012, leg. Jian-Kun Long, Wei-Bin Zheng, Shi-Yan Xu; 8♂♂ 8♀♀, Guizhou Province, Suiyang County, Kuankuoshui National Nature Reserve (28°14'N, 107°12'E), 2–4 June 2010, leg. Yan-Li Zheng; 10♂♂ 6♀♀, Guizhou Province, Tongren City, Fanjingshan National Nature Reserve (27°55'N, 108°42'E), 20–24 September 2011, leg. Wei-Bin Zheng, Zhi-Min Chang, Xiao-Fei Yu, Zhi-Hua Fan; 17♂♂ 4♀♀, Guizhou Province, Tongren City, Fanjingshan National Nature Reserve, Yinjiang County, Yongyi Township, Dayuanzhi Village, (27°54'N, 108°38'E), 29 May 2002, leg. Xiang-Sheng Chen; 1♂, Guizhou Province, Tongren City, Fanjingshan National Nature Reserve, Heihewan (27°50'N, 108°46'E), 30 July 2014, leg. Meng-Shu Dong; 1♀, Guizhou Province, Tongren City, Fanjingshan National Nature Reserve, Jinding (27°54'N, 108°42'E), 1 September 1996, leg. Mao-Fa Yang; 3♀♀, Guizhou Province, Tongren City, Fanjingshan National Nature Reserve, Jinding, 30 July 2001, leg. Mao-Fa Yang, Guo-Dong Ren; 4♂♂ 6♀♀, Guizhou Province, Leishan County, Leigongshan National Forest Park (26°21'N, 108°9'E), 4–6 July 2011, leg. Wei-Bin Zheng, Jian-Kun Long; 3♂♂ 2♀♀, Guizhou Province, Leishan County, Leigongshan National Forest Park, Lianhuaping, 31 May–3 June 2005, leg. Zi-Zhong Li, Qiong-Zhang Song, Bin Zhang; 5♂♂ 4♀♀, Guizhou Province, Duyun City, Chachang (26°24'N, 107°36'E), 12 May 2014, leg. Ming Ning, Gai-Ping Yang, Ding-Guo Li; 2♂♂ 1♀, Guizhou Province, Duyun City, Chachang, 16 August 2014, leg. Gai-Ping Yang, Ding-Guo Li; 1♂ 4♀♀, Guizhou Province, Wangmo County, Dayi Town (25°10'N, 106°06'E), 22 August 2012, leg. Shi-Yan Xu, Wei-Bin Zheng; 1♂ 1♀, Guizhou Province, Guiyang City, Guizhou Botanical Garden (26°37'N, 106°44'E), 18 June 2008, leg. Jun-Qiang Ni; 3♂♂ 3♀♀, Guizhou Province, Guiyang City, Wudang District (26°38'N, 106°45'E), 5 June 2009, leg. Qiong-Zhang Song; 1♂ 2♀♀, Guizhou Province, Zunyi City, Loushanguan (28°1'N, 106°51'E), 21 September 2017, leg. Bin Yan; 15♂♂ 3♀♀, Guizhou Province, Xishui County, Linjiang (28°19'N, 106°12'E), 1 June 2006, leg. Xiang-Sheng Chen.

#### Supplementary description.

**Figure 8.**
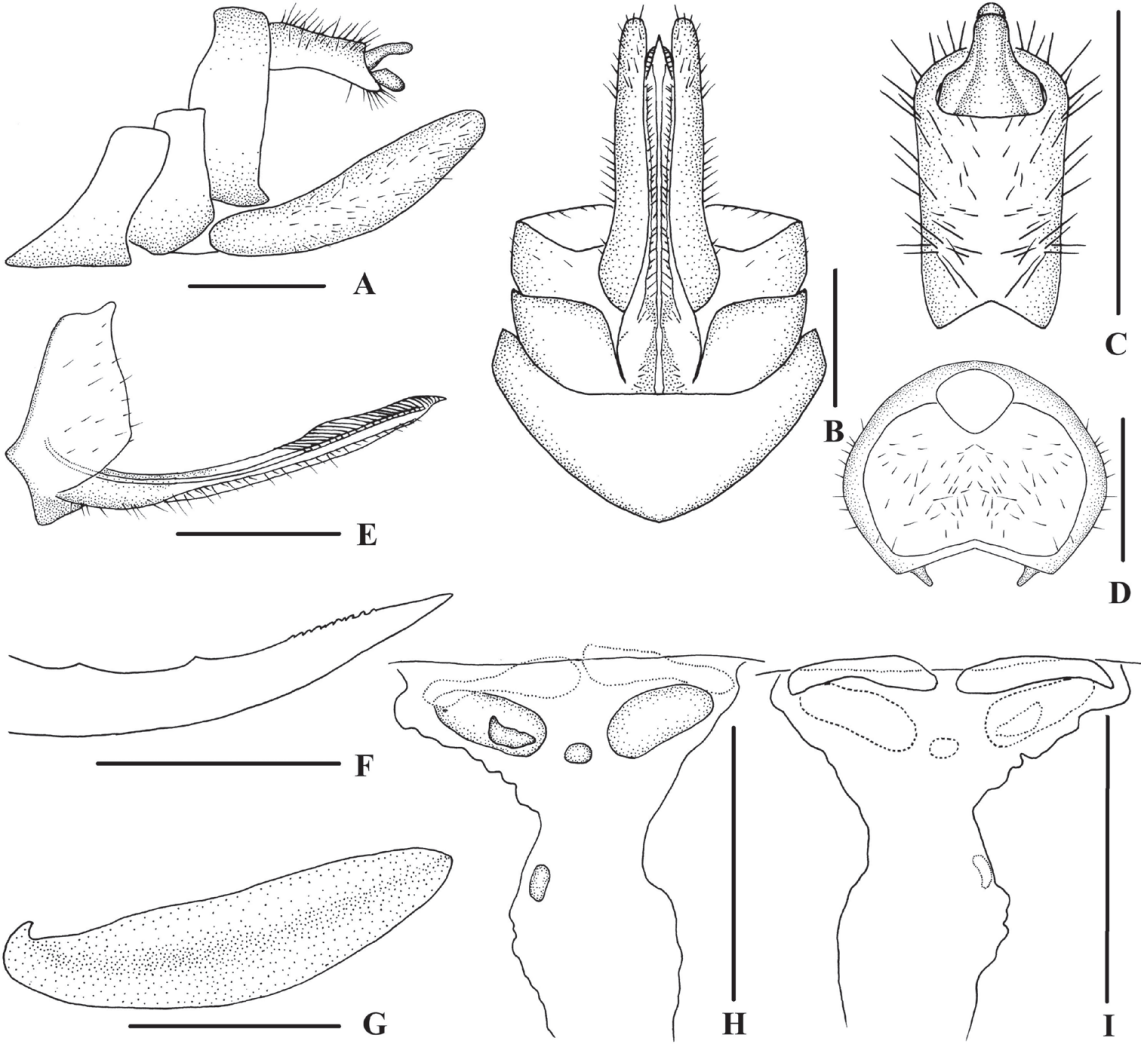
*Kuveraussuriensis* (Vilbaste, 1968), female **A** genitalia, lateral view **B** genitalia, ventral view **C** anal segment, dorsal view **D** tergite IX, caudal view **E** gonapophysis VIII and gonocoxa VIII, ventral view **F** gonapophysis IX, lateral view **G** gonoplac, inner lateral view **H** posterior vagina, ventral view **I** posterior vagina, dorsal view. Scale bars: 0.5 mm.

***Female genitalia*.** Tergite IX (Fig. 8A, B, D) moderately sclerotized, with a large wax plate, nearly oval, dorsal and ventral margins concave. Anal segment (Fig. 8C) rectangular, 1.8 times wider than long in dorsal view, anal style strap-like. Gonapophysis VIII (Fig. 8E) elongate, and slightly curved upwards. Gonapophysis IX (Fig. 8F) with two middle teeth, distance ratio between distal middle tooth to apex and length of denticulate portion is 2.9. Gonoplac (Fig. 8G) rod-like, 4.0 times longer than wide in lateral view. Posterior vagina pattern as shown in Figure 8H, I.

**Figure 9.**
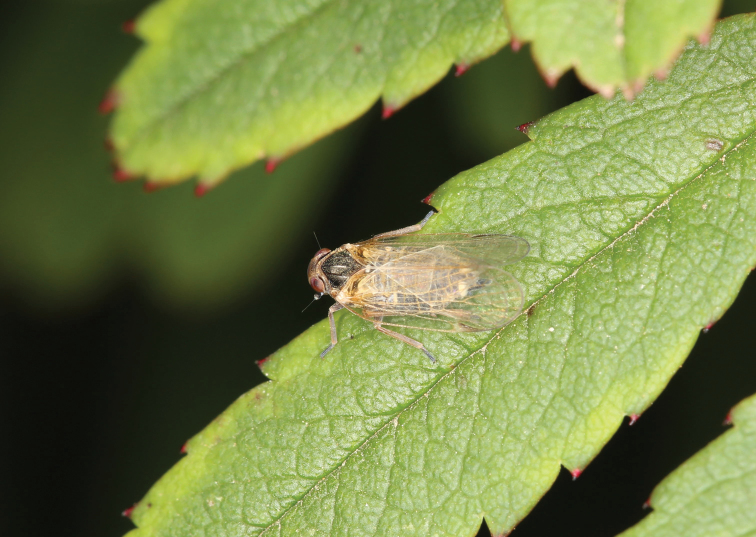
Adult of *Kuveraussuriensis* (Vilbaste, 1968), dorsal view, female (Caohai National Natural Reserve, Weining County, Guizhou Province, 2 August 2017, photograph by Xiang-Sheng Chen).

#### Host plant.

**Figure 10.**
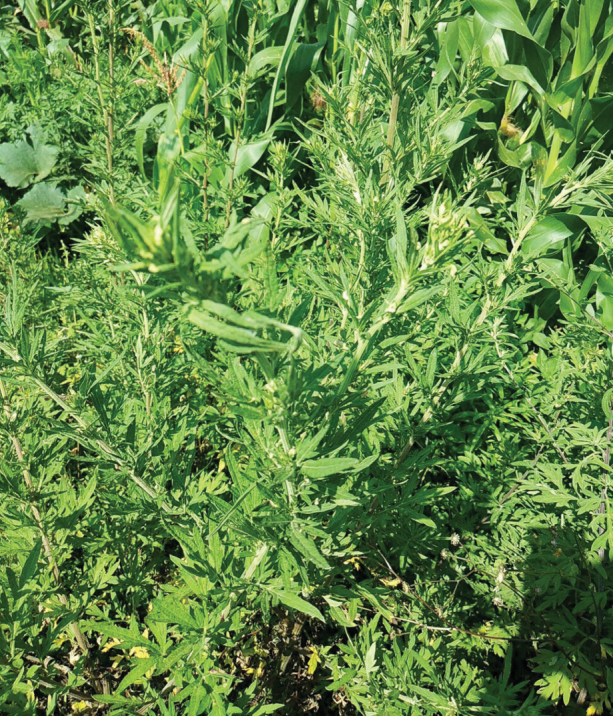
The habitat of *Kuveraussuriensis* (Vilbaste, 1968) (3 August 2017, Caohai National Natural Reserve, Weining County, Guizhou Province, photograph by Yan Zhi).

*Artemisiamongolica* (Fisch. ex Bess.) Nakai (Asteraceae) (Fig. 10).

#### Distribution.

China (Anhui, Guangxi, Guizhou, Hebei, Hunan, Shaanxi, Shanxi, Sichuan, Yunnan), Japan, Russia.

#### Remarks.

This species can be distinguished from other species of the genus by the following characters: anal segment symmetrical; aedeagus with three processes: left spinose process of periandrium long, gently curved and apex directed left-ventrocephalad; ventral surface of periandrium with a spinose process, slightly curved and apex directed right-cephalad; spinose process of endosoma stout and long, nearly reaching apex of endosoma.

#### Note.

The female genitalia of this species are described and illustrated for the first time.

## ﻿Discussion

The biology of few *Kuvera* species throughout the world are well-known. The plant associations of genus have been reported in several previous studies (Anufriev 1987; Emeljanov 2015; Luo et al. 2019). In this study, we found *Kuveraussuriensis* (Vilbaste, 1968) on *Artemisiamongolica* (Fisch. ex Bess.) Nakai.

**Figure 11.**
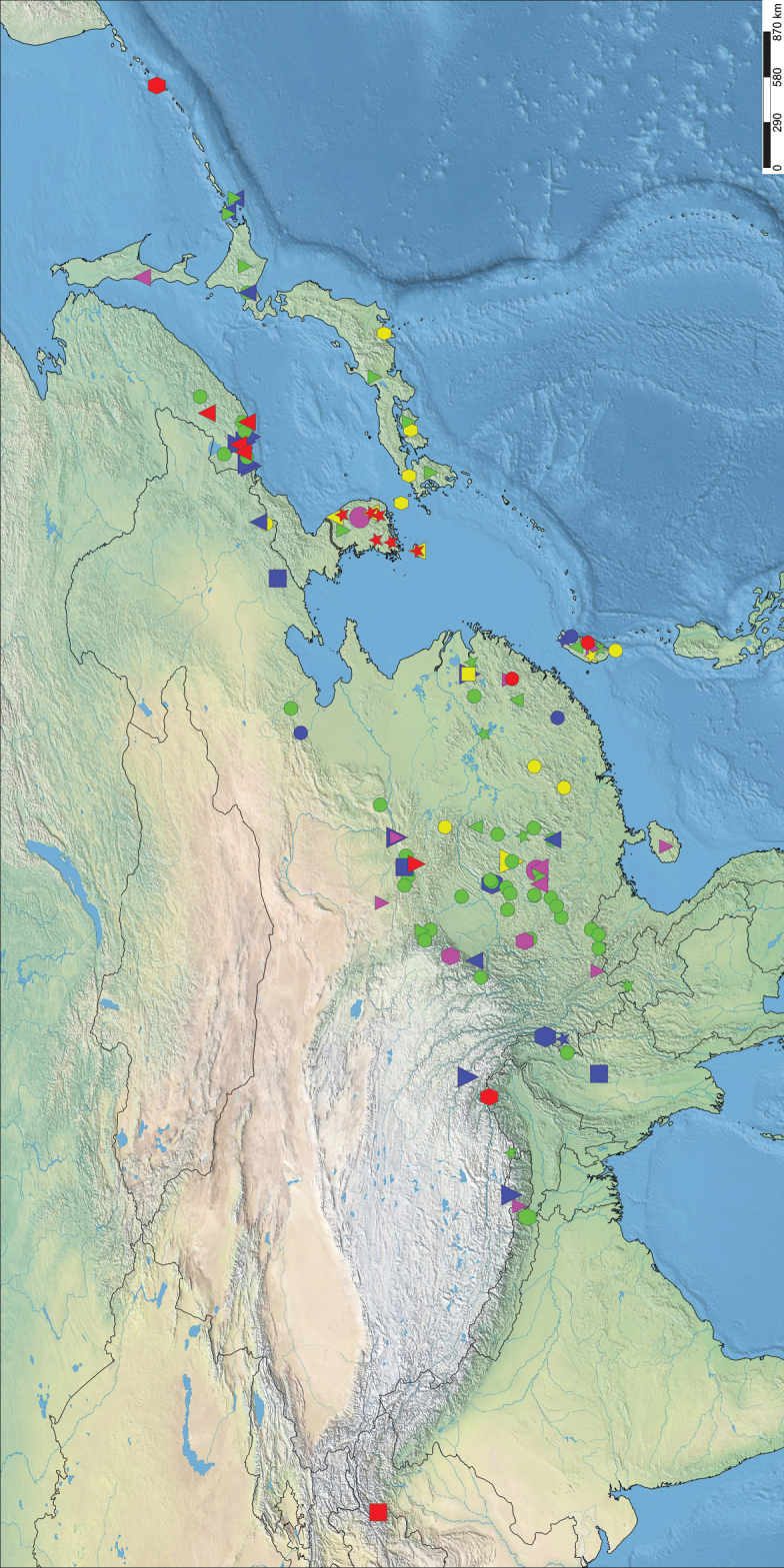
Distribution records of species from the genus *Kuvera: K. amurensis* (red triangle); *K.basarukini* (pink triangle); *K.brunettii* (green hexagon); *K.brunnea* (red square); *K.campylotropa* sp. nov. (blue hexagon); *K.communis* (red circle); *K.elongata* sp. nov. (yellow inverse triangle); *K.flaviceps* (green inverse triangle); *K.hagilsanensis* (red star); *K.hallasanensis* (yellow triangle); *K.hama* (green triangle); *K.huoditangensis* (red inverse triangle); *K.kurilensis* (red hexagon); *K.laticeps* (pink hexagon); *K.ligustri* (yellow hexagon); *K.longipennis* (yellow star); *K.longwangshanensis* (yellow square); *K.pallidula* (blue triangle); *K.semihyalina* (blue square); *K.similis* (blue circle); *K.taiwana* (pink inverse triangle); *K.tappanella* (yellow circle); *K.toroensis* (green star); *K.transversa* (blue star); *K.ussuriensis* (green circle); *K.vilbastei* (blue inverse triangle); and *K.yecheonensis* (pink circle).

Based on data from published information and our field surveys, the distribution records of all twenty-seven known species of *Kuvera* was summarized in Figure 11. Up to now, the genus presents a distribution pattern in the Palearctic, Sino-Japanese, and Oriental regions. Compared with Luo et al. (2019), new distribution records of several species have been added recently (Luo et al. 2022 and this study). We believe that the actual distribution range of most species is still unclear. *Kuverabrunettii* Muir, 1922, *K.brunnea* (Dlabola, 1957), *K.hagilsanensis* Rahman, Kwon & Suh, 2017, *K.hallasanensis* Rahman, Kwon & Suh, 2017, *K.ligustri* Matsumura, 1914, *K.longipennis* Matsumura, 1914 and *K.longwangshanensis* Luo, Liu & Feng, 2019 are known only from the type locality, and further collecting and investigation of this genus are still needed. The complex and variable geomorphological environment and rich biological resources of the distribution area create a variety of habitat types, which are likely reasons for the rich species diversity of the genus *Kuvera*.

## Supplementary Material

XML Treatment for Kuvera

XML Treatment for Kuvera basarukini

XML Treatment for Kuvera campylotropa

XML Treatment for Kuvera elongata

XML Treatment for Kuvera laticeps

XML Treatment for Kuvera ussuriensis
